# Transmission of Severe Acute Respiratory Syndrome Coronavirus 2 during Border Quarantine and Air Travel, New Zealand (Aotearoa)

**DOI:** 10.3201/eid2705.210514

**Published:** 2021-05

**Authors:** Nick Eichler, Craig Thornley, Tara Swadi, Tom Devine, Caroline McElnay, Jillian Sherwood, Cheryl Brunton, Felicity Williamson, Josh Freeman, Sarah Berger, Xiaoyun Ren, Matt Storey, Joep de Ligt, Jemma L. Geoghegan

**Affiliations:** Auckland District Health Board, Auckland, New Zealand (N. Eichler, F. Williamson);; Hutt Valley District Health Board, Lower Hutt, New Zealand (C. Thornley); New Zealand Ministry of Health, Wellington, New Zealand (T. Swadi, T. Devine, C. McElnay); Institute of Environmental Science and Research, Porirua, New Zealand (J. Sherwood, X. Ren, M. Storey, J. de Ligt, J.L. Geoghegan); Canterbury District Health Board, Christchurch, New Zealand (C. Brunton, J. Freeman, S. Berger); University of Otago, Dunedin, New Zealand (J.L. Geoghegan)

**Keywords:** severe acute respiratory syndrome coronavirus 2, SARS-CoV-2, coronavirus, coronavirus disease, COVID-19, viruses, border quarantine, air travel, case-patients, respiratory infections, zoonoses, New Zealand

## Abstract

The strategy in New Zealand (Aotearoa) to eliminate coronavirus disease requires that international arrivals undergo managed isolation and quarantine and mandatory testing for severe acute respiratory syndrome coronavirus 2. Combining genomic and epidemiologic data, we investigated the origin of an acute case of coronavirus disease identified in the community after the patient had spent 14 days in managed isolation and quarantine and had 2 negative test results. By combining genomic sequence analysis and epidemiologic investigations, we identified a multibranched chain of transmission of this virus, including on international and domestic flights, as well as a probable case of aerosol transmission without direct person-to-person contact. These findings show the power of integrating genomic and epidemiologic data to inform outbreak investigations.

New Zealand (Aotearoa in Māori) has a goal of eliminating coronavirus disease (COVID-19), which has resulted in a low incidence of this disease in this country (*1*–*3*). Managed isolation and quarantine (MIQ) is the mainstay of postborder controls to minimize importation risk. With few exceptions, international arrivals to New Zealand undergo a mandatory 14-day period of MIQ in designated facilities before entering the community. MIQ facilities are repurposed commercial hotels used exclusively for isolation and quarantine of returnees.

During the MIQ period, regular health monitoring, as well as PCR testing on days 3 and 12, is undertaken to identify persons with COVID-19, whether symptomatic or asymptomatic, and measures are taken to control transmission. Subsequent to this study, a day 1 test has also been put in place, as have predeparture tests. Persons who complete their 14-day period, show negative PCR results for severe acute respiratory syndrome coronavirus 2 (SARS-CoV-2), and remain asymptomatic are cleared to be released. We report a case of COVID-19 in a recent arrival to New Zealand in September 2020.

## Human Ethics

A review by the New Zealand Health and Disability Ethics Committees advised that that its approval was not required for this study. Nasopharyngeal samples that had positive results for SARS-CoV-2 by real-time reverse transcription PCR were obtained from public health medical diagnostics laboratories located throughout New Zealand. Under contract for the Ministry of Health, the Institute of Environmental Science and Research has approval to conduct genomic sequencing for surveillance of notifiable diseases.

## Index Case-Patient

On September 18, 2020, a COVID-19 case was identified in New Zealand. The case was in a person who was a recent international arrival from India who had completed 14 days in MIQ in Christchurch, New Zealand, had shown negative results twice for SARS-CoV-2 on days 3 and 12, and had subsequently been released. This case-patient is denoted as case-patient G.

Case-patient G flew from Christchurch to Auckland, New Zealand, on the day of release on a government-chartered flight with several other persons released from MIQ. This case-patient subsequently showed development of symptoms and showed positive results for SARS-CoV-2 four days later. Persons who had close contact with case-patient G were subsequently monitored and tested (Table). All persons who were positive for SARS-CoV-2 as a result of this investigation have provided verbal consent to be included in this study.

**Table:** Characteristics for 9 case-patients tested for transmission of severe acute respiratory syndrome coronavirus 2 during border quarantine and air travel, New Zealand, September, 2020*

Case-patient	Symptom onset date	Positive sample date	Probable source of infection	Place of probable acquisition	GISAID accession no.	Flight seating details		
						India to Fiji: Aug 26	Fiji to Christchurch: Aug 27	Christchurch to Auckland: Sep 11
A	Asymptomatic	Aug 30	Residence overseas	India	EPI_ISL_548116	Row 50–55	7D	19A
B	Aug 29	Aug 30	Case-patient A or same source as case-patient A	In India or during travel to New Zealand	EPI_ISL_548118	53A	19D	Not on flight
C	Sep 6	Sep 8	Case-patients A or B	During travel to New Zealand	EPI_ISL_579092	49D	10F	Not on flight
D	Asymptomatic	Sep 21	Case-patient C	MIQ	EPI_ISL_579108	NR	17C	5A
E	Asymptomatic	Sep 21	Case-patient D	MIQ (child of case-patient D)	EPI_ISL_579105	NR	17C	5A
F	Sep 22	Sep 21	Case-patient E	Household (parent of case-patient E)	EPI_ISL_579107	Not on flight	Not on flight	Not on flight
G	Sep 15	Sep 17	Case-patient D	Domestic flight from Christchurch to Auckland	EPI_ISL_579103	55G	18F	4A
H	Sep 17	Sep 19	Case-patient G	Household (partner of case-patient G)	EPI_ISL_579104	Not on flight	Not on flight	Not on flight
I	Asymptomatic	Sep 19	Case-patient D	Household (child of case-patients G and H)	EPI_ISL_579099	Not on flight	Not on flight	Not on flight

*MIQ, managed isolation and quarantine; NR, not reported.

## Travel from India to New Zealand

Case-patient G had been part of a cohort of 149 repatriated New Zealand citizens or permanent residents who had returned from India to New Zealand on August 27, 2020. The entire cohort who arrived in Christchurch had traveled on the same chartered flight (a Boeing 747) from Delhi, India, through Nadi, Fiji; all passengers disembarked from the flight in Fiji. Several passengers remained in Fiji, 3 of whom later showed positive results for SARS-CoV-2 during their quarantine period but who were not included in this investigation. Predeparture testing for SARS-CoV-2 was not mandatory at the time and no passengers reported having been tested.

**Figure 1:**
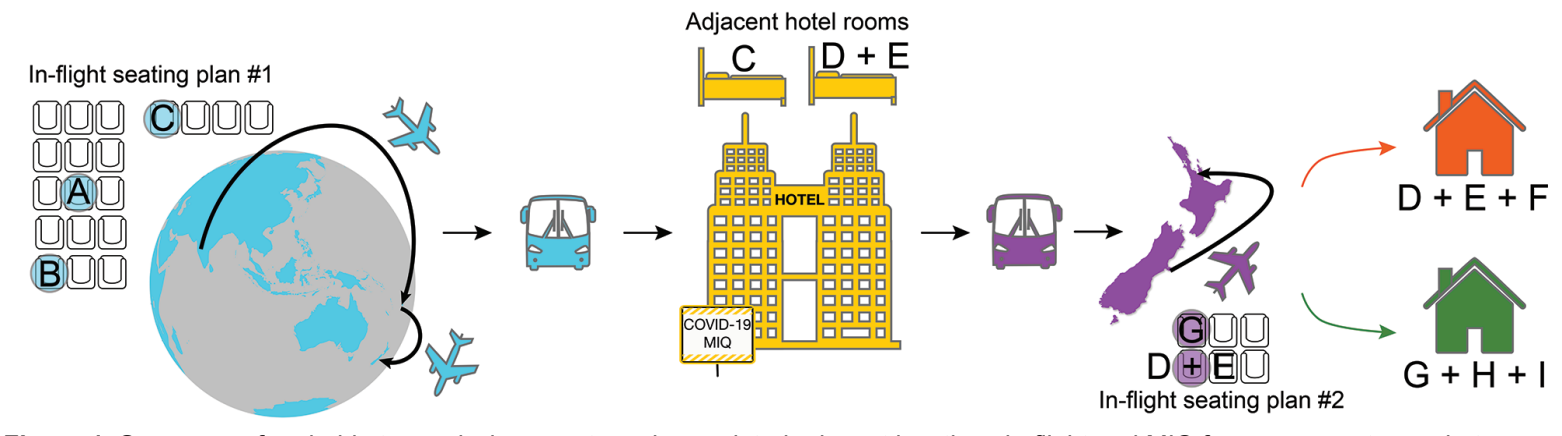
Sequence of probable transmission events and associated relevant locations in-flight and MIQ for severe acute respiratory syndrome coronavirus 2 during border quarantine and air travel, New Zealand, September 2020. Location of case A is approximate (Table). COVID-19, coronavirus disease; MIQ, managed isolation and quarantine.

Of the persons who arrived in Christchurch on this flight, 8 showed positive results for SARS-CoV-2 while in MIQ. Of these 8 case-patients, 3 were shown to be genomically linked and are denoted as case-patients A, B, and C (Figure 1). During the first (≈18 hour) flight from New Delhi to Nadi, case-patients A, B, and C sat within 2 rows of each other; all other case-patients observed physical distancing (Table). The flight was at ≈35% occupancy, and passengers were evenly spaced throughout the aircraft.

The timing at which case-patient C experienced symptoms was consistent with transmission during the flight from India to New Zealand by case-patient A or B. Case-patients A or B might have been infected during or before the flight from a common source. All passengers were required to wear facemasks for the duration of the flight, and the flight crew followed infection prevention measures. The passengers in question did not travel together and did not know each other. On arrival in Christchurch, passengers were disembarked in groups of 10 to enable physical distancing to be maintained in the terminal, and each case-patient was provided with a fresh surgical mask. The cohort was transferred by bus to MIQ upon arrival in Christchurch. Physical distancing and surgical mask use were used while boarding and on board, but seating was not preallocated to specific passengers.

## Evidence of Transmission in Hotel-Managed Isolation and Quarantine

The MIQ facility was a repurposed commercial hotel, in which each room had its own bathroom and no balconies. Case-patient C was positive on day 12 and was relocated to the isolation section of the facility. Before their relocation, an adult and infant child, both of whom had returned from India on the same flight, were in the adjacent room (Figure 1). Both the adult and child completed their 14-day quarantine. Each person had 2 negative test results and no reported symptoms but later showed positive results for SARS-CoV-2 while in the community (these 2 case-patients are denoted as case-patients D and E). We consider that these 2 case-patients were infected while in MIQ.

Closed-circuit television review of the period between the arrival of case-patients C, D, and E and the transfer of case-patient C to the isolation section of MIQ showed that there were no instances where the 3 persons were outside of their rooms at the same time. Nevertheless, footage showed that during routine testing on day 12, which took place within the doorway of the hotel rooms, there was a 50-second window between closing the door to the room of case-patient C and opening the door to the room of case-patients D and E. Therefore, we hypothesized that suspended aerosol particles were the probable mode of transmission in this instance, and that the enclosed and unventilated space in the hotel corridor probably facilitated this event (*4*). A commissioned review of the ventilation system found that the rooms in question had a net positive pressure compared with the corridor. Fomite transmission through use of communal bins in the corridor was considered to be a less probable route of transmission because contact with the bin lid by case-patient D was >20 hours after it was touched by case-patient C.

## Domestic In-Flight and Household Transmission

Following their 14-day completion of MIQ, case-patients A (who was deemed to be recovered), D, E, and G boarded an 85-min government-chartered domestic flight (on a Boeing 737) from Christchurch to Auckland. All passengers were required to wear masks, and the flight was at ≈50% occupancy. Case-patient G sat directly in front of case-patients D and E, and case-patient A sat at a distance (Figure 1). On arrival at Auckland airport, case-patients D and E were met by a household contact, denoted as case-patient F, and case-patient G was met by household contacts (case-patients H and I). These household contacts had not been in MIQ because they had no recent history of travel outside New Zealand. However, both contacts subsequently tested positive for SARS-CoV-2 (Figure 1).

## Genome Sequencing of SARS-CoV-2

**Figure 2:**
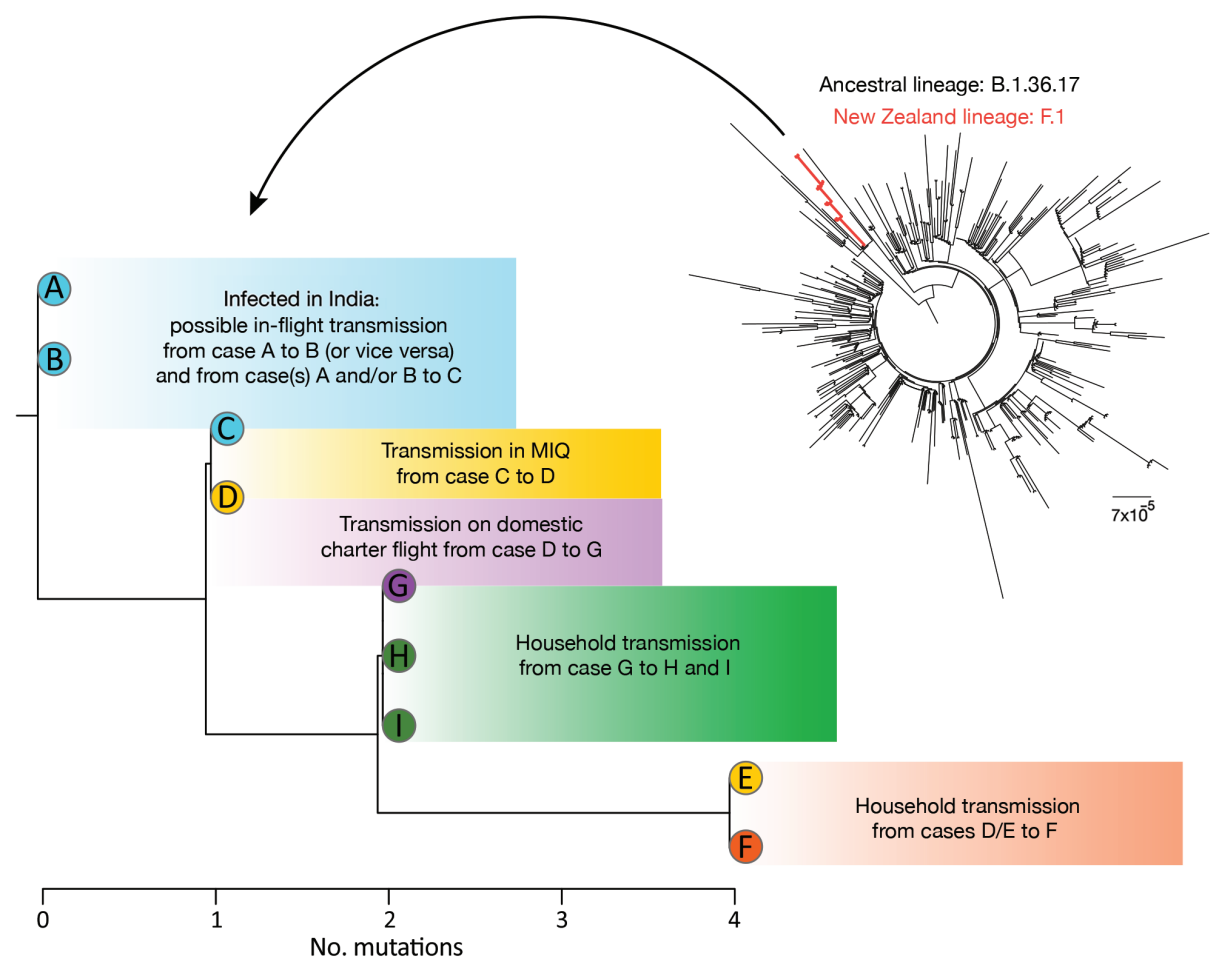
Phylogenetic trees showing genomic relationship of severe acute respiratory syndrome coronavirus 2 genomes generated for 9 case-patients, New Zealand, September 2020. Shown are number of mutations, as well as the F.1 cluster (red) within the context of the closest ancestral B.1.36.17 lineage (black). Scale bar indicates nucleotide substitutions per site. MIQ, managed isolation and quarantine.

We generated the genomes of the 9 positive SARS-CoV-2 samples from case-patients A–I according to reported sequencing protocols (*5*–*7*) (https://github.com/ESR-NZ/NZ_SARS-CoV-2_genomics). These genomes were classified within the (now ancestral) PANGO (*8*) genomic lineage B.1.36.17. Because of the dynamic nature of this genomic nomenclature, this cluster from New Zealand is now classified as lineage F.1, which is now extinct (Figure 2).

We compared these data to virus genomes sequenced from New Zealand and those B.1.36.17 genomes from the global dataset that were available on GISAID (https://www.gisaid.org) as of February 2021 (n = 1,994) (*9*). The 9 SARS-CoV-2 sequences from New Zealand, together with 500 B.1.36.17 genomes, uniformly sampled at random from the global population (Appendix), were aligned by using MAFFT version 7 and the FFT-NS-2 algorithm (*10*). Ambiguous sites that have been flagged as potential sequencing errors were masked. We created a maximum-likelihood phylogenetic tree by using IQ-TREE version 1.6.8 (*11*) and the Hasegawa-Kishino-Yano (*12*) nucleotide substitution model with a gamma-distributed rate variation among sites. We determined the best fit model by using ModelFinder (*13*). We assessed branch support by using the ultrafast bootstrap method (*14*).

We found a genomic link between virus isolated from all 9 case-patients and a maximum genomic distance of 4 single-nucleotide polymorphisms (Figure 2). Placing this cluster within the global context provides high confidence (100% bootstrap node support of 1,000 iterations) that it was a single introduction of the virus into New Zealand (Figure 2). Of the other 5 case-patients who were positive for SARS-CoV-2 and arrived on the same flight from India, 1 case-patient was definitively excluded from the cluster on the basis of virus genome being within a different (non-F.1) genomic PANGO lineage (Appendix). Four samples did not contain adequate RNA for genomic sequencing.

## Conclusions

This case study of COVID-19 transmission demonstrates a multibranched chain of transmission involving numerous settings, supported by closed-circuit television observations, genomic sequence analyses, and epidemiologic investigations. Major aspects included a probable case of transmission without direct person-to-person contact by aerosol within MIQ; transmission in-flight, as well as within households; and use of genomic sequence analysis to confirm probable direction of transmission between cases. These findings reinforce the need for rigorous border control processes for countries pursuing COVID-19 elimination, as well as real-time integration of genomic and epidemiologic data to inform outbreak investigations.

Appendix Additional information on transmission of severe acute respiratory syndrome coronavirus 2 during border quarantine and air travel, New Zealand.
